# Delayed Diagnosis of Pancreatic Tail Cancer Presenting With Chronic Scrotal Pain

**DOI:** 10.7759/cureus.24756

**Published:** 2022-05-05

**Authors:** Morika Suzuki, Takashi Watari

**Affiliations:** 1 Department of General Internal Medicine, National Hospital Organization Sendai Medical Center, Miyagi, JPN; 2 General Medicine Center, Shimane University Hospital, Izumo, JPN; 3 Division of Hospital Medicine, University of Michigan Health System, Ann Arbor, USA

**Keywords:** seminal metastasis, delayed diagnosis, diagnostic error, scrotal pain, malignant pancreatic cancer

## Abstract

In most cases, diagnosing pancreatic cancer (PC) in a timely manner is challenging owing to the lack of a specific set of symptoms, especially for cancer originating in the pancreatic tail. A patient presented to our hospital with the primary complaint of bilateral scrotal pain caused by metastatic pancreatic tail cancer. Although he had a family history of PC, there were no other risk factors. Here, we discuss the challenges associated with early diagnosis of malignant PC, particularly when it presents in the tail of the organ, and spermatic cord metastasis caused by pancreatic tail cancer.

## Introduction

Pancreatic cancer (PC) is difficult to detect due to a lack of specific symptoms. Diagnosis of pancreatic tail cancer is particularly difficult because it is less common, less likely to cause jaundice than cancer of the pancreatic head, and difficult to detect on abdominal ultrasound [1]. Risk factors include a family history of PC and a personal history of diabetes and smoking [2]. Testicular metastases from PC are rare [3].

Chronic scrotal pain (CSP) refers to constant or intermittent testicular pain for three months or longer that interferes with daily activities [4]. CSP can be due to pain from the scrotal contents directly or due to referred pain from the abdomen/inguinal region, retroperitoneum, or nervous system [5,6]. However, in many cases, no source is identified [5,6].

A 75-year-old man with a family history of PC presented with CSP, which was subsequently found to be due to testicular metastases of pancreatic tail cancer. We present this case with a review of the literature.

## Case presentation

A 75-year-old man with a history of chronic low back pain and benign prostatic hyperplasia presented with bilateral scrotal pain that had gradually worsened from intermittent to continuous over the previous three months. He also reported a dull ache in his lower abdomen. On examination, his vital signs were normal. Physical examination of the scrotum revealed no erythema, masses, or tenderness. His abdomen was flat, soft, and mildly tender on palpation. Bilateral lower abdominal pressure resulted in exacerbated scrotal pain. He reported mild spontaneous pain in the lumbar region, which was not exacerbated by compression, positioning, or straight leg raising. No abnormalities were detected on routine blood tests or abdominal ultrasound, and lumbar spine magnetic resonance imaging (MRI) was negative for spinal disease.

**Figure 1:**
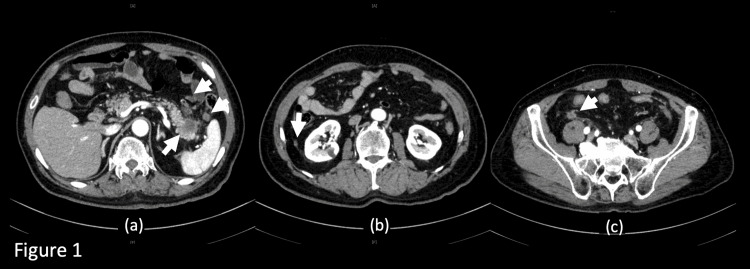
Enhanced computed tomography of the abdomen. (a) A 3-cm mass in the pancreatic tail (long arrow) with lymph node enlargement (short arrows). (b-c) Several nodules (arrows) believed to be peritoneal metastases of pancreatic cancer.

Ten days later, the patient presented with increased abdominal pain, and he disclosed that he had a younger sister with PC. Abdominal computed tomography (CT) revealed an ill-defined, poorly contrasting, 3-cm mass with indistinct borders in the pancreatic tail, enlarged lymph nodes, and numerous nodules in the pelvis, suggestive of peritoneal dissemination (Figure 1).

There were no noticeable mass lesions in the scrotum or spermatic cord. The patient was diagnosed with pancreatic tail cancer with peritoneal dissemination, and chemotherapy was initiated. The primary lesion decreased in size, and the scrotal pain improved; however, the dose of chemotherapy was reduced due to pancytopenia, and the pancreatic tumor and scrotal pain worsened. Due to cancerous peritonitis and paralytic ileus, the patient had difficulty eating and died seven months after the initial presentation.

## Discussion

This patient had advanced pancreatic tail cancer with peritoneal metastases at the time of the initial presentation. This case illustrates the difficulty in diagnosing pancreatic tail cancer, as well as the difficulty in identifying the source of CSP caused by the metastasis of PC.

PC is difficult to detect due to a lack of specific symptoms. Furthermore, 78% of PCs occur in the head of the pancreas and, when accompanied by jaundice due to biliary obstruction, are detected early. In contrast, pancreatic tail cancer is more difficult to detect as it is less common (22%), less likely to cause jaundice, and difficult to visualize by abdominal ultrasound [1]. Approximately one-quarter of patients with PC experience abdominal discomfort for six months before being diagnosed, and approximately half of patients report back pain at the initial visit [1]. Fatigue, changes in bowel habits, weight loss, and decreased appetite can result in earlier PC diagnoses [7]. This patient did not complain of these symptoms at the initial visit, but it was later found that his appetite was lower than usual, and he experienced fatigue after he became aware of scrotal pain. A retrospective study of patients with PC found that 31.3% of patients were not correctly diagnosed at the initial visit when they were initially diagnosed with gallbladder disease or reflux esophagitis [8].

In Japan, approximately 7% of patients with PC have a family history of PC [2], which is similar to that reported in Western countries (7-10%). The risk of PC increases as the number of close relatives with PC increases, and the lifetime incidence rate of PC is higher in patients with a family history of PC [9]. In this case, the patient had a first-degree relative with PC who had developed the disease at a younger age, and he reported abdominal discomfort at the time of the initial visit, making it necessary to consider PC in the differential diagnosis. However, we did not suspect PC at the initial visit because the patient did not report any common symptoms of PC, such as jaundice, recent onset of back pain, or weight loss; did not have a suggestive history or lifestyle risk factors such as diabetes or smoking history; and did not disclose his family history of PC. Furthermore, the patient’s chief presenting complaint at the initial visit was CSP, which made the diagnosis more difficult.

The reason for the low incidence of secondary tumors to the testis is the low scrotal temperature. It is thought that this may impair the ability of tumor cells from solid organs to metastasize [10]. Metastatic carcinoma to the testis may present with a palpable mass in the scrotum or cause scrotal pain, which may lead to CSP. Testicular metastases are relatively common in prostate and gastric cancers but are rare in pancreatic cancer, and the route of metastasis is not clearly understood [10-12]. Various routes of spread of metastatic tumors to the testes have been proposed, including direct spread along the vas deferens to the epididymis, transperitoneal seeding through a patent tunica vaginalis, retrograde lymphatic spread, retrograde venous spread, and arterial embolization [3,12,13]. In this case, we believe that the patient experienced CSP due to transperitoneal seeding of PC through a patent tunica vaginalis.

## Conclusions

This case discussed a 75-year-old patient with spermatic cord metastasis due to pancreatic tail cancer who presented to our hospital with the chief complaint of CSP. Pancreatic tail carcinoma is particularly prone to delayed diagnosis, and its metastatic manifestations are diverse. As in this case, testicular metastasis of tumors, including PC, should be considered in the differential diagnosis of scrotal pain.
